# Syntenic gene analysis between *Brassica rapa* and other Brassicaceae species

**DOI:** 10.3389/fpls.2012.00198

**Published:** 2012-08-30

**Authors:** Feng Cheng, Jian Wu, Lu Fang, Xiaowu Wang

**Affiliations:** Institute of Vegetables and Flowers, Chinese Academy of Agricultural SciencesBeijing, China

**Keywords:** synteny, ortholog, *Brassica rapa*, *Arabidopsis thaliana*, *Arabidopsis lyrata*, *Thellugiella parvula*, Brassicaceae

## Abstract

Chromosomal synteny analysis is important in genome comparison to reveal genomic evolution of related species. Shared synteny describes genomic fragments from different species that originated from an identical ancestor. Syntenic genes are orthologs located in these syntenic fragments, so they often share similar functions. Syntenic gene analysis is very important in Brassicaceae species to share gene annotations and investigate genome evolution. Here we designed and developed a direct and efficient tool, SynOrths, to identify pairwise syntenic genes between genomes of Brassicaceae species. SynOrths determines whether two genes are a conserved syntenic pair based not only on their sequence similarity, but also by the support of homologous flanking genes. Syntenic genes between *Arabidopsis thaliana* and *Brassica rapa*, *Arabidopsis lyrata* and *B. rapa*, and *Thellungiella parvula* and *B. rapa* were then identified using SynOrths. The occurrence of genome triplication in *B. rapa* was clearly observed, many genes that were evenly distributed in the genomes of *A. thaliana*, *A. lyrata*, and *T. parvula* had three syntenic copies in *B. rapa*. Additionally, there were many *B. rapa* genes that had no syntenic orthologs in *A. thaliana*, but some of these had syntenic orthologs in *A. lyrata* or *T. parvula*. Only 5,851 genes in *B. rapa* had no syntenic counterparts in any of the other three species. These 5,851 genes could have originated after *B. rapa* diverged from these species. A tool for syntenic gene analysis between species of Brassicaceae was developed, SynOrths, which could be used to accurately identify syntenic genes in differentiated but closely-related genomes. With this tool, we identified syntenic gene sets between *B. rapa* and each of *A. thaliana*, *A. lyrata*, *T. parvula*. Syntenic gene analysis is important for not only the gene annotation of newly sequenced Brassicaceae genomes by bridging them to model plant *A. thaliana*, but also the study of genome evolution in these species.

## Introduction

The genomes of species from Brassicaceae are composed of 24 basic genomic blocks, A–X (24 GBs, also called the ancestral karyotypes, AK) (Schranz et al., 2006), as detected by studies using comparative chromosomal painting (CCP) (Mandakova and Lysak, 2008), and also directly observed in *Arabidopsis thaliana* (Initiative, 2000), and the newly sequenced genomes of *Arabidopsis lyrata* (Hu et al., 2011), *Brassica rapa* (Wang et al., 2011), and *Thellungiella parvula* (Dassanayake et al., 2011). Brassicaceae genomes have experienced different levels of genomic reshuffling, fragmentation, deletion, segmental or whole genome duplication (Schranz et al., 2006; Mandakova and Lysak, 2008). Along with drastic genome changes, gene contents have also rapidly evolved (Cheng et al., 2012; Tang et al., 2012). However, as they share a common AK, the chromosomal synteny relationship among these genomes is considered to be well preserved despite the long time for evolution following the divergence of these species (Tang et al., 2008; Cheng et al., 2012).

Chromosomal synteny analysis is important in genome comparison to reveal the genomic evolution of related species (Tang et al., 2008). Shared synteny describes genomic fragments from different species that originated from a certain common ancestor (Lyons et al., 2008). Syntenic genes are orthologs located in these syntenic fragments, so they often share similar functions, and we can be highly confident when sharing their functional annotation information.

Determining syntenic genes and genomic regions among closely related species is important to both gene and genome studies (Lyons et al., 2008). On one hand, with a well-defined synteny relationship, we can share gene annotation information between well-studied genomes (such as the model species *A. thaliana*) and newly annotated genomes (such as *B. rapa*), and investigate the syntenic gene's differentiation after the species' divergence. On the other hand, syntenic fragments shared among genomes of different species offer us a chance to deduce the evolutionary processes of related species, or could even provide clues to the mechanisms behind these processes.

Three plant species, *B. rapa*, *B. juncea*, and *B. oleracea*, (also called genomes A, B, and C) and their allopolyploidies form the famous U's triangle (U, 1935). Many of these are important vegetable or oil crop species, and the sequencing of more genomes from these species is being planned. The A genome sequence of *B. rapa* has been released (Wang et al., 2011), and compared to three other sequenced species from Brassicaceae (*A. thaliana*, *A. lyrata*, and *T. parvula*), *B. rapa* has experienced an extra genome triplication event after their divergence (Wang et al., 2011; Cheng et al., 2012).

To share gene annotations between *B. rapa* and the other annotated genomes specially for *A. thaliana* and dig for clues of the *B. rapa* genome evolution, we developed a tool, SynOrths, which is well suited for detecting syntenic orthologs between closely related species, and applied it to the synteny analysis between *B. rapa* and other sequenced genomes from Brassicaceae: the model plant *A. thaliana*, *A. lyrata*, and *T. parvula*. The datasets generated in this study will help the gene annotation and further genomic evolution analysis of these species.

## Results

### Development of the synorths tool to identify gene pairs with syntenic relationships

**Figure 1:**
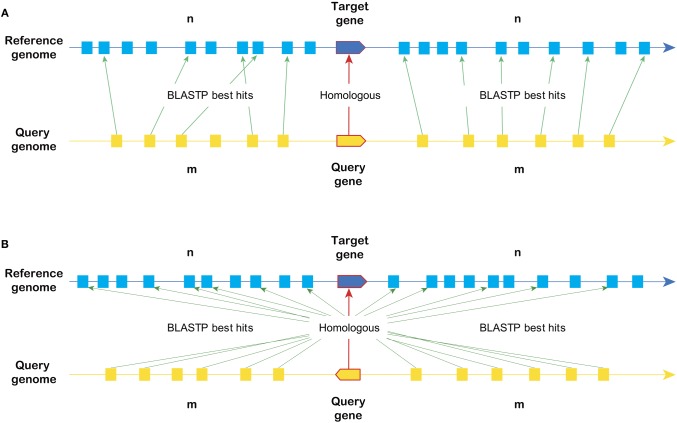
**The principles of syntenic gene identification in SynOrths.** When determining whether two genes are under synteny, both the sequence homology of the two genes themselves and their flanking genes are considered. **(A)** Syntenic genes in the same direction in each genome. **(B)** Two syntenic genes located in inverted syntenic fragments.

A tool named SynOrths was developed to identify syntenic genes based on the protein sequences of *B. rapa* and other related species (http://brassicadb.org/brad/tools/SynOrths/). As shown in Figure 1, SynOrths determines two genes to be syntenic orthologs based on both their own sequence similarity and the homology of their flanking genes.

There are four main steps embedded in SynOrths: (1) finding orthologous gene pairs; (2) redundant tandem gene removal; (3) locating potential syntenic orthologs by the support of flanking genes; and (4) final syntenic gene pair determination. In the first step, SynOrths runs Blastp to get basic protein sequence homology information from pairwise genomes. Gene pairs that are the best hits or with Blastp e-values <1E-20 are selected for further analysis. For tandem duplicated genes, which would add complexity to syntenic gene finding, SynOrths keeps one gene from each tandem gene array as a representative. In the second step, we identify all tandem gene arrays across the genomes being compared. Each tandem array is composed of continuously distributed homologous genes (Blastp E-value <1E-20) and should not be interrupted by more than one non-homologous gene. After that, the genes of each tandem array are replaced by the first one in the corresponding tandem. The revised homologous gene pairs are then sent to step 3 to compute the supporting strength of the flanking genes. Here, we set a threshold to check if the gene pair in question is supported by their flanking genes and thus potentially syntenic. Genes located in both flanking regions of the two genes are selected and named as the flanking gene set. We then count the number of best hit genes between the pairwise genomes in the flanking gene set. If the ratio of the best hit genes is higher than the threshold, then the homologous gene pair is considered as potentially syntenic. In the fourth step, we further screen for the best syntenic gene pairs. After the first three steps, a certain gene might have more than one potential syntenic partner, so these candidate syntenic gene pairs are then compared based on the ratio of flanking gene support and their sequence homology. The gene pair with the highest supporting ratio of flanking genes and comparably higher sequence homology is finally determined as the best syntenic pair.

**Figure 2:**
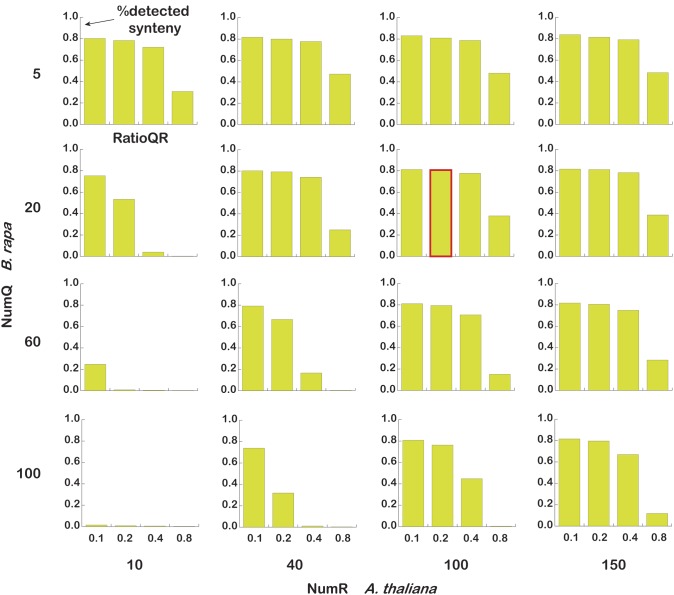
**Parameter estimation in SynOrths.** The number of query (*B. rapa*) flanking genes [5, 20, 60, 100], the number of reference (*A. thaliana*) flanking genes [10, 40, 100, 150], and the threshold of the flanking genes' support ratio [0.1, 0.2, 0.4, 0.8] were set to run SynOrths. The bars indicate the proportions of syntenic genes identified out of 38,161 *B. rapa* genes, “%detected synteny” means percent of identified syntenic genes to the 38,161 *B. rapa* genes. The bar with a red border is the run with parameters NumQ = 20, NumR = 100, and RatioQR = 0.2; SynOrths returned stable and relatively more syntenic genes under these parameters.

There are three main parameters that should be considered when using SynOrths. They are NumQ, the number of flanking genes on each side of the query gene; NumR, the number of flanking genes on each side of the reference gene; RatioQR, the ratio of best hit pairs among these flanking genes. Because *B. rapa* experienced whole genome triplication and subsequent intensive gene loss, the parameters should be carefully selected when using SynOrths to determine its syntenic genes with other species. Here, we chose three arrays for the three parameters (NumQ [5, 20, 60, 100], NumR [10, 40, 100, 150], and RatioQR [0.1, 0.2, 0.4, 0.8]) to perform SynOrths analysis between *B. rapa* and *A. thaliana*. As shown in Figure 2, the parameters NumQ = 20, NumR = 100, RatioQR = 0.2 gave a considerably better result. This parameter set was then chosen to identify syntenic gene pairs between *B. rapa* and *A. thaliana*, *A. lyrata*, and *T. parvula*.

### Syntenic gene determination between *B. rapa* and each of *A. thaliana*, *A. lyrata*, and *T. parvula*

Syntenic gene pairs between *B. rapa* and *A. thaliana*, *B. rapa* and *A. lyrata*, and *B. rapa* and *T. parvula* were identified using SynOrths. There were a total of 41,174, 27,379, 33,410, and 28,910 annotated proteins for *B. rapa*, *A. thaliana*, *A. lyrata*, and *T. parvula*, respectively. After removing the redundancy of duplicated tandem genes (keeping one gene from each tandem array), 38,161, 24,939, 30,773, and 27,344 genes were left for syntenic gene determination (Table 1). *B. rapa* returned 30,615 genes syntenic to 18,410 genes in *A. thaliana*; 30,250 genes syntenic to 18,125 *A. lyrata* genes; and 29,473 genes syntenic to 17,303 *T. parvula* genes. *A. thaliana* had the highest syntenic gene ratio (80.1%) compared to *A. lyrata* (79.6%) and *T. parvula* (77.5%).

**Table 1:** **The homologous relationships of genes between *B. rapa* and *A. thaliana*, *A. lyrata*, or *T. parvulla***.

	**Total genes**	**Tandem redundancy removed**	**Syntenic orthologs to *B. rapa*^a^**	**Non-syntenic orthologs to *B. rapa*^b^**	**Non-orthologs**
*B. rapa*	41,174	38,161	N	N	N
*A. thaliana*	27,379	24,939	18,410/30,615	2,561/1,391	3,968/6,155
*A. lyrata*	33,410	30,773	18,125/30,250	1,877/1,226	10,771/6,685
*T. parvulla*	28,901	27,344	17,303/29,473	3,909/1,605	6,132/7,083
*At* + *Al* + *Tp*	N	N	N/32,310	N/808	N/5,043

a Numbers left of the ‘/’ indicate gene numbers in At, Al, or Tp; numbers right of the ‘/’ represent the gene numbers in Br.

b Non-syntenic orthologs were defined as gene pairs with sequence identity >70% and coverage >60%.

**Figure 3:**
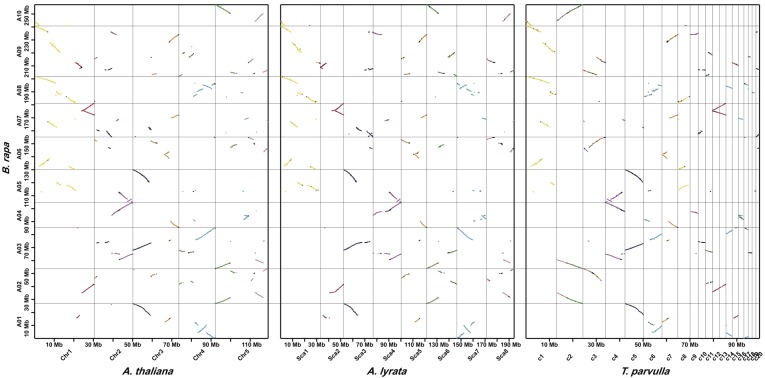
**Syntenic genes identified by SynOrths between *B. rapa* and *A. thaliana*, *A. lyrata*, or *T. parvula*.** For each segment in *A. thaliana*, *A. lyrata*, or *T. parvula*, there were three syntenic copies observed in *B. rapa*, which clearly reflected the genome triplication experienced by *B. rapa*. Colors of the dots represent for the 24 ancestral blocks of Brassicaceae species, which has been defined previously (Schranz et al., 2006).

The genome triplication event in *B. rapa* was well supported (Figure 3), because many genes that were evenly distributed in genomes of *A. thaliana* (14.3%), *A. lyrata* (14.6%), and *T. parvula* (16.1%) had three syntenic copies in *B. rapa*. Additionally, among the 7,546 *B. rapa* genes that had no syntenic orthologs in *A. thaliana*, 849 were syntenic to genes in *A. lyrata*, and 1,416 syntenic to *T. parvulla* genes. In total, there were 32,310 *B. rapa* genes with at least one syntenic ortholog in either *A. thaliana*, *A. lyrata*, or *T. pavula*, and only 5,851 *B. rapa* genes that had no syntenic counterparts in any of the other species.

For these non-syntenic genes between *B. rapa* and the other species, we considered them non-syntenic orthologs if their similarity satisfied sequence identity >70%, and coverage for each of the two genes >60%. *B. rapa* returned 1,391, 1,226, and 1,605 non-syntenic orthologs to 2,561 genes in *A. thaliana*, 1,877 in *A. lyrata*, 3,909 in *T. parvula*, respectively. However, for the 5,851 genes that had no syntenic orthologs in all three species, only 808 genes were non-syntenic orthologs of at least one gene in the other three species. These 808 genes could have been generated by gene transposition in *B. rapa* after its divergence from *A. thaliana*, *A. lyrata*, and *T. parvula*.

Most of the tandem arrays in *B. rapa* showed a syntenic relationship to *A. thaliana*, *A. lyrata*, or *T. parvula* (Table 2). For all 2,137 tandem arrays in *B. rapa*, 1,649 (77.16%) were syntenic to *A. thaliana*; 1,751 (81.94%) syntenic to *A. lyrata*, and 1,689 (79.04%) syntenic to *T. parvula*. In total, 1,864 (87.23%) tandem arrays in *B. rapa* had syntenic counterparts in at least one of the other species.

**Table 2:** **Syntenic tandem genes between *B. rapa* and *A. thaliana*, *A. lyrata*, or *T. parvulla***.

	**Tandem (#arrays|#genes)**	**Syntenic tandem to *B. rapa*^a^**
*B. rapa*	2,137|5,150	N
*A. thaliana*	1,569|4,009	1,223|3,157/1,649|4,033
*A. lyrata*	1,751|4,388	1,204|3,098/1,751|4,267
*T. parvulla*	1,135|2,692	857|2,071/1,689|4,140
*At* + *Al* + *Tp*	N|N	N|N/1,864|4,542

a Numbers left of the ‘/’ indicate tandems in At, Al, or Tp; numbers right of the ‘/’ represent tandem numbers in Br.

The dataset of genes' syntenic relationship among above four species had been integrated into BRAD (Brassica Database, http://brassicadb.org/brad/searchBrMultiSynteny.php) (Cheng et al., 2011). This resource built bridges between model plant *A. thaliana* and other Brassicaceae species, so the information of genes' function studies in *A. thaliana* were linked to the newly sequenced and annotated Brassicaceae genomes. For crop species such as *B. rapa, B. oleracea*, and *B. napus*, with the resource of syntenic relationships we can rapidly transfer knowledges from *A. thaliana* to the breeding research and apllication, and further production of the crops.

## Discussion

Because SynOrths determines syntenic orthologs and genomic regions based on protein sequences, it cannot be applied to genomes without protein annotation. Coding regions are one of the most conserved elements in the genome, as most active genes are functionally conserved to defend against non-synonymous mutations. Even after long periods of divergent evolution, proteins with the same function still show high levels of sequence homology. Thus, proteins that preserve ancestral traits provide good data for synteny analysis. After the determination of syntenic orthologs, syntenic gene pairs could be used as anchors to identify genomic regions that are in synteny. Conversely, most non-coding genomic regions (except conserved non-coding DNA) are under almost neutral selection. These regions can be rapidly changed in the evolutionary process, even in true syntenic regions non-coding sequences almost always show complete non-homology. SynOrths is not suited for synteny analysis of genomes without protein sets, but methods that include a step to search for conserved non-coding elements for synteny analysis might handle this problem.

The performance of SynOrths is impacted by gene annotation; the more accurate the gene annotation sets, the more accurately syntenic orthologs would be identified. However, the impact is acceptable and can even be very limited when we tune down the parameters to deny gene pairs to be in synteny. Because the annotation of different genomes is completed by different software with different parameters, the robustness of synteny analysis is very important. Furthermore, where the annotation of different genomes is unbalanced, we cannot draw genetic relationships based on the number or ratio of identified syntenic genes. Syntenic gene sets could be used to address whole genome genetic variation patterns, but for research issues focusing on individual genes, more detailed work should be done to confirm the results, or the parameters should be turned up to generate more stringent results.

Synteny analysis is one of the most important fields in comparative genome analysis as it is the basis of evolutionary studies at both the gene and genome levels. Most practically, it helps improve the gene annotation of newly sequenced genomes. In this study, we used synteny analysis to link bulk gene information from the model plant *A. thaliana* to the newly sequenced Brassicaceae genomes *B. rapa*, *A. lyrata*, and *T. parvula*.

We designed and developed a direct and efficient tool, SynOrths, to identify pairwise syntenic genes between *B. rapa* and other sequenced genomes of Brassicaceae species. With this tool we conducted syntenic gene analysis between *B. rapa* and *A. thaliana*, *A. lyrata*, and *T. parvula*, and generated valuable datasets for further analysis. Whole genome synteny analysis and the datasets generated are important for both gene annotation of the newly sequenced Brassicaceae genomes and the study of genomic and chromosomal evolution in Brassicaceae species.

## Materials and methods

Gene annotation and protein sequences of *B. rapa* version 1.2 were downloaded from BRAD (http://brassicadb.org) (Cheng et al., 2011). The *A. thaliana* dataset was retrieved from TAIR (http://www.arabidopsis.org/index.jsp), and version TAIR9 was used. Genome and gene annotation files of version 6 for *A. lyrata* were downloaded from the JGI database (http://genome.jgi-psf.org/Araly1/Araly1.home.html) (Hu et al., 2011). *T. parvula* datasets version 7 were obtained from the *T. parvula* genome sequencing group (Dassanayake et al., 2011).

Blastp (NCBI-BLAST package, ftp://ftp.ncbi.nih.gov/blast/) was used for protein sequence alignment in SynOrths. SynOrths now is freely available at http://brassicadb.org/brad/tools/SynOrths/. The data generated in this study can be searched through http://brassicadb.org/brad/searchBrMultiSynteny.php or by contacting Feng Cheng.

## Author contributions

Xiaowu Wang and Feng Cheng conceived the study. Feng Cheng developed SynOrths and performed the synteny analysis. Feng Cheng prepared the manuscript, Xiaowu Wang and Jian Wu improved the manuscript. Jian Wu and Lu Fang tested SynOrths and provided feedback. All authors read and approved the final manuscript.

### Conflict of interest statement

The authors declare that the research was conducted in the absence of any commercial or financial relationships that could be construed as a potential conflict of interest.
